# Supplementary material for: Hot spot prediction in protein-protein interactions by an ensemble system
Source: BMC Syst Biol. 2018 Dec 31;12(Suppl 9):132. doi: 10.1186/s12918-018-0665-8 (PMC6311905; doi:10.1186/s12918-018-0665-8)
Supplement: Supplementary file 2 — All relevant MATLAB code. A simple Matlab implement of our predictor. (ZIP 19,742 kb) [file 12918_2018_665_MOESM2_ESM.zip › Additional file 2/Read ASA/ASA website/ASEdb/1NMBH.htm]

 NetSurfP - Protein Surface Accessibility and Secondary Structure Predictions

  

|  |  |  |
| --- | --- | --- |
|  |  | NetSurfP - Protein Surface Accessibility and Secondary Structure Predictions  Technical University of Denmark |

  


---

```
# For publication of results, please cite:
# A generic method for assignment of reliability scores applied to solvent accessibility predictions.
# Bent Petersen, Thomas Nordahl Petersen, Pernille Andersen, Morten Nielsen and Claus Lundegaard
# BMC Structural Biology 2009, 9:51 doi:10.1186/1472-6807-9-51
#
# Column 1: Class assignment - B for buried or E for Exposed - Threshold: 25% exposure, but not based on RSA
# Column 2: Amino acid
# Column 3: Sequence name
# Column 4: Amino acid number
# Column 5: Relative Surface Accessibility - RSA
# Column 6: Absolute Surface Accessibility
# Column 7: Z-fit score for RSA prediction
# Column 8: Probability for Alpha-Helix
# Column 9: Probability for Beta-strand
# Column 10: Probability for Coil
E Q  1NMBH                  1    0.707 126.217   1.174   0.005   0.015   0.979
B V  1NMBH                  2    0.187  28.665  -0.247   0.004   0.616   0.381
E Q  1NMBH                  3    0.373  66.618   1.262   0.002   0.816   0.182
B L  1NMBH                  4    0.126  23.071   0.752   0.018   0.846   0.136
E Q  1NMBH                  5    0.328  58.509   0.763   0.021   0.756   0.223
B Q  1NMBH                  6    0.202  36.024   0.522   0.022   0.552   0.426
B P  1NMBH                  7    0.351  49.750  -0.354   0.021   0.279   0.699
E G  1NMBH                  8    0.411  32.338  -1.654   0.018   0.088   0.893
E A  1NMBH                  9    0.496  54.703  -2.193   0.018   0.047   0.935
E E  1NMBH                 10    0.477  83.279  -1.600   0.019   0.141   0.840
E L  1NMBH                 11    0.333  61.046  -0.693   0.022   0.359   0.619
E V  1NMBH                 12    0.301  46.325   0.376   0.022   0.359   0.619
E K  1NMBH                 13    0.384  78.948   0.952   0.020   0.205   0.775
E P  1NMBH                 14    0.442  62.663  -0.744   0.018   0.047   0.935
E G  1NMBH                 15    0.581  45.709  -2.111   0.018   0.047   0.935
E A  1NMBH                 16    0.408  44.984  -0.936   0.004   0.085   0.910
E S  1NMBH                 17    0.383  44.946  -0.663   0.005   0.336   0.660
B V  1NMBH                 18    0.142  21.825   0.736   0.003   0.718   0.279
E R  1NMBH                 19    0.366  83.768   0.662   0.001   0.900   0.099
B M  1NMBH                 20    0.067  13.447   0.545   0.001   0.900   0.099
B S  1NMBH                 21    0.112  13.103   0.529   0.001   0.900   0.099
B C  1NMBH                 22    0.034   4.830   0.085   0.001   0.900   0.099
B K  1NMBH                 23    0.248  51.014   0.570   0.001   0.900   0.099
B A  1NMBH                 24    0.081   8.926  -0.400   0.003   0.718   0.279
B S  1NMBH                 25    0.147  17.252  -0.453   0.004   0.420   0.576
B G  1NMBH                 26    0.192  15.095  -1.826   0.004   0.138   0.858
B Y  1NMBH                 27    0.239  51.160  -1.018   0.021   0.279   0.699
B T  1NMBH                 28    0.145  20.070  -0.884   0.021   0.451   0.528
B F  1NMBH                 29    0.092  18.484  -0.723   0.021   0.451   0.528
B T  1NMBH                 30    0.188  26.048  -1.224   0.021   0.279   0.699
B N  1NMBH                 31    0.255  37.288  -1.245   0.019   0.141   0.840
B Y  1NMBH                 32    0.092  19.746   0.194   0.019   0.141   0.840
B N  1NMBH                 33    0.069  10.102   0.033   0.021   0.451   0.528
B M  1NMBH                 34    0.023   4.682   0.641   0.021   0.756   0.223
B Y  1NMBH                 35    0.042   8.975  -0.381   0.018   0.846   0.136
B W  1NMBH                 36    0.030   7.167   0.509   0.018   0.846   0.136
B V  1NMBH                 37    0.032   4.934   0.240   0.018   0.846   0.136
B K  1NMBH                 38    0.111  22.750   0.399   0.021   0.756   0.223
B Q  1NMBH                 39    0.079  14.092   0.381   0.022   0.359   0.619
B S  1NMBH                 40    0.193  22.655   0.681   0.005   0.045   0.951
E P  1NMBH                 41    0.323  45.777   0.696   0.005   0.015   0.979
E G  1NMBH                 42    0.525  41.333  -1.457   0.018   0.019   0.964
E Q  1NMBH                 43    0.400  71.440   0.586   0.018   0.019   0.964
E G  1NMBH                 44    0.433  34.077  -0.300   0.018   0.088   0.893
B L  1NMBH                 45    0.149  27.319   0.975   0.005   0.336   0.660
B E  1NMBH                 46    0.097  16.911  -0.329   0.003   0.718   0.279
B W  1NMBH                 47    0.042  10.077  -0.019   0.001   0.900   0.099
B I  1NMBH                 48    0.022   4.051   1.103   0.001   0.959   0.040
B G  1NMBH                 49    0.016   1.243   0.151   0.001   0.959   0.040
B I  1NMBH                 50    0.074  13.671  -0.584   0.001   0.959   0.040
B F  1NMBH                 51    0.019   3.773   1.212   0.001   0.959   0.040
B Y  1NMBH                 52    0.143  30.580   0.749   0.003   0.718   0.279
B P  1NMBH                 53    0.270  38.370  -0.934   0.005   0.262   0.733
E G  1NMBH                 54    0.551  43.403  -2.041   0.005   0.015   0.979
E N  1NMBH                 55    0.556  81.413  -2.586   0.016   0.005   0.979
E G  1NMBH                 56    0.509  40.090  -2.262   0.018   0.019   0.964
E D  1NMBH                 57    0.451  64.960  -0.563   0.018   0.088   0.893
B T  1NMBH                 58    0.276  38.295   0.032   0.021   0.279   0.699
E S  1NMBH                 59    0.399  46.763   0.165   0.021   0.279   0.699
B Y  1NMBH                 60    0.201  42.954   0.395   0.020   0.205   0.775
B N  1NMBH                 61    0.183  26.864  -0.531   0.052   0.084   0.864
E Q  1NMBH                 62    0.618 110.464  -0.523   0.053   0.043   0.903
E K  1NMBH                 63    0.398  81.766   0.125   0.018   0.088   0.893
B F  1NMBH                 64    0.077  15.494  -0.330   0.020   0.205   0.775
E K  1NMBH                 65    0.366  75.368   0.918   0.004   0.197   0.799
B D  1NMBH                 66    0.196  28.186  -1.558   0.005   0.336   0.660
B K  1NMBH                 67    0.200  41.078   0.826   0.002   0.816   0.182
B A  1NMBH                 68    0.044   4.882   0.107   0.001   0.900   0.099
B T  1NMBH                 69    0.102  14.120   0.694   0.001   0.959   0.040
B L  1NMBH                 70    0.036   6.592   0.637   0.001   0.959   0.040
B T  1NMBH                 71    0.094  13.066   0.543   0.002   0.816   0.182
B A  1NMBH                 72    0.221  24.376   0.517   0.004   0.514   0.481
B D  1NMBH                 73    0.242  34.829  -0.157   0.004   0.420   0.576
E K  1NMBH                 74    0.437  89.932   0.093   0.018   0.088   0.893
B S  1NMBH                 75    0.216  25.303  -1.893   0.018   0.047   0.935
E S  1NMBH                 76    0.515  60.323   0.953   0.018   0.088   0.893
B N  1NMBH                 77    0.247  36.102  -0.294   0.021   0.279   0.699
B T  1NMBH                 78    0.177  24.508   0.997   0.021   0.756   0.223
B A  1NMBH                 79    0.060   6.645  -0.370   0.011   0.918   0.071
B Y  1NMBH                 80    0.150  32.098   0.517   0.011   0.918   0.071
B M  1NMBH                 81    0.042   8.364   0.673   0.011   0.918   0.071
B Q  1NMBH                 82    0.117  20.986  -0.283   0.018   0.846   0.136
B L  1NMBH                 83    0.064  11.700  -0.023   0.022   0.552   0.426
E S  1NMBH                 84    0.314  36.789  -0.179   0.021   0.279   0.699
E S  1NMBH                 85    0.355  41.653  -0.820   0.019   0.141   0.840
B L  1NMBH                 86    0.095  17.303   0.236   0.018   0.088   0.893
E T  1NMBH                 87    0.457  63.344   1.041   0.018   0.047   0.935
E S  1NMBH                 88    0.341  39.918  -0.529   0.053   0.043   0.903
E E  1NMBH                 89    0.407  71.033  -0.579   0.053   0.043   0.903
B D  1NMBH                 90    0.148  21.269  -0.841   0.053   0.043   0.903
B S  1NMBH                 91    0.097  11.345   0.443   0.022   0.359   0.619
B A  1NMBH                 92    0.031   3.471  -0.188   0.018   0.846   0.136
B V  1NMBH                 93    0.058   8.884   0.952   0.011   0.918   0.071
B Y  1NMBH                 94    0.029   6.112   1.091   0.011   0.918   0.071
B Y  1NMBH                 95    0.041   8.804   0.992   0.018   0.846   0.136
B C  1NMBH                 96    0.013   1.853   1.702   0.021   0.756   0.223
B A  1NMBH                 97    0.024   2.623   0.521   0.023   0.655   0.322
B R  1NMBH                 98    0.082  18.755   0.192   0.005   0.336   0.660
B S  1NMBH                 99    0.228  26.663  -0.582   0.018   0.088   0.893
B G  1NMBH                100    0.246  19.329  -1.040   0.018   0.047   0.935
E G  1NMBH                101    0.388  30.520  -2.356   0.018   0.047   0.935
E S  1NMBH                102    0.318  37.305  -2.818   0.018   0.047   0.935
B Y  1NMBH                103    0.267  57.015  -2.738   0.018   0.047   0.935
B R  1NMBH                104    0.229  52.510  -2.204   0.018   0.047   0.935
B Y  1NMBH                105    0.247  52.720  -1.688   0.018   0.047   0.935
B D  1NMBH                106    0.302  43.533  -2.183   0.018   0.019   0.964
B G  1NMBH                107    0.213  16.802  -1.081   0.018   0.047   0.935
B G  1NMBH                108    0.089   6.996  -0.951   0.018   0.088   0.893
B F  1NMBH                109    0.042   8.349  -0.304   0.019   0.141   0.840
B D  1NMBH                110    0.059   8.444  -0.886   0.019   0.141   0.840
B Y  1NMBH                111    0.048  10.364  -0.287   0.020   0.205   0.775
B W  1NMBH                112    0.038   9.091  -0.348   0.019   0.141   0.840
B G  1NMBH                113    0.035   2.762  -1.935   0.004   0.138   0.858
B Q  1NMBH                114    0.128  22.807   0.013   0.004   0.138   0.858
B G  1NMBH                115    0.081   6.335  -1.893   0.005   0.262   0.733
B T  1NMBH                116    0.076  10.513   0.223   0.004   0.514   0.481
E T  1NMBH                117    0.253  35.133   0.193   0.002   0.816   0.182
B L  1NMBH                118    0.047   8.569   0.503   0.001   0.900   0.099
B T  1NMBH                119    0.133  18.447  -0.015   0.001   0.900   0.099
B V  1NMBH                120    0.106  16.215  -0.586   0.002   0.816   0.182
E S  1NMBH                121    0.290  33.953  -0.829   0.004   0.514   0.481
E S  1NMBH                122    0.602  70.543  -2.098   0.003   0.003   0.994

Explain the output.  Go back.
```

---
